# Sclerostin and Vascular Pathophysiology

**DOI:** 10.3390/ijms21134779

**Published:** 2020-07-06

**Authors:** Antonino Catalano, Federica Bellone, Nunziata Morabito, Francesco Corica

**Affiliations:** 1 Department of Clinical and Experimental Medicine, University of Messina, 98125 Messina, Italy; fbellone@unime.it (F.B.); nmorabito@unime.it (N.M.); coricaf@unime.it (F.C.); 2 A.O.U. Policlinico “G.Martino”, Via Consolare Valeria, 98125 Messina, Italy

**Keywords:** sclerostin, Wnt, cardiovascular, atherosclerosis, aging, calcification, chronic kidney disease, diabetes mellitus

## Abstract

There is cumulating evidence for a contribution of Wnt signaling pathways in multiple processes involved in atherosclerosis and vascular aging. Wnt signaling plays a role in endothelial dysfunction, in the proliferation and migration of vascular smooth muscle cells (VSMCs) and intimal thickening. Moreover, it interferes with inflammation processes, monocyte adhesion and migration, as well as with foam cell formation and vascular calcification progression. Sclerostin is a negative regulator of the canonical Wnt signaling pathway and, accordingly, the consequence of increased sclerostin availability can be disruption of the Wnt signalling cascade. Sclerostin is becoming a marker for clinical and subclinical vascular diseases and several lines of evidence illustrate its role in the pathophysiology of the vascular system. Sclerostin levels increase with aging and persist higher in some diseases (e.g., diabetes, chronic kidney disease) that are known to precipitate atherosclerosis and enhance cardiovascular risk. Current knowledge on the association between sclerostin and vascular diseases is summarized in this review.

## 1. Introduction

Aging produces profound effects on the vasculature, and it is strictly associated with the development cardiovascular and cerebrovascular diseases, which are the most common causes of death among the elderly in developed countries [1]. The circulatory system may influence the local environment of tissues and organs. Therefore, age-induced functional and structural alterations of microcirculation play a role in the pathophysiology of a wide range of known age-related disorders, including cognitive impairment, sarcopenia and kidney or eye diseases [1,2].

Several aging mechanisms have been variously considered as contributing to the pathogenesis of both microvascular and macrovascular diseases such as oxidative stress, mitochondrial dysfunction, impaired resistance to molecular stressors, chronic low-grade inflammation, genomic instability, telomeric attrition and cellular senescence, epigenetic alterations, stem cell exhaustion and altered intercellular communication [3].

Since several age-related cardiovascular and cerebrovascular diseases arise from alterations in arterial function, or are worsened by arterial functional and phenotypic changes, elucidating the basic mechanisms underlying arterial aging seems to be a matter of interest.

In the last decades, signalling triggered by the Wnt family of secreted glycoproteins has emerged as one of the major mechanisms associated with a plethora of biological processes including embryonic and adult stem cell development, cell differentiation, proliferation, polarity and migration [4,5,6,7]. The term “Wnt” is a portmanteau word derivied from the blend of the name of the Wingless segment polarity gene in drosophila and that of its vertebrate homolog, known as integrated or int-1. These conserved signalling pathways begin with proteins transmitting to the cell through its surface receptors [5].

As emerged from loss- and gain-of-function experimental models, Wnt signalling might contribute to vascular development and homeostasis [8].

In this review, we first illustrate the function of Wnt signalling and, secondly, we highlight the role of this pathway in vascular pathophysiology. The available evidence from in vitro and in vivo studies about Wnt proteins, receptors and antagonists, with a special focus on sclerostin, are shown below.

## 2. Wnt Signaling Pathways: A Synopsis

**Figure 1:**
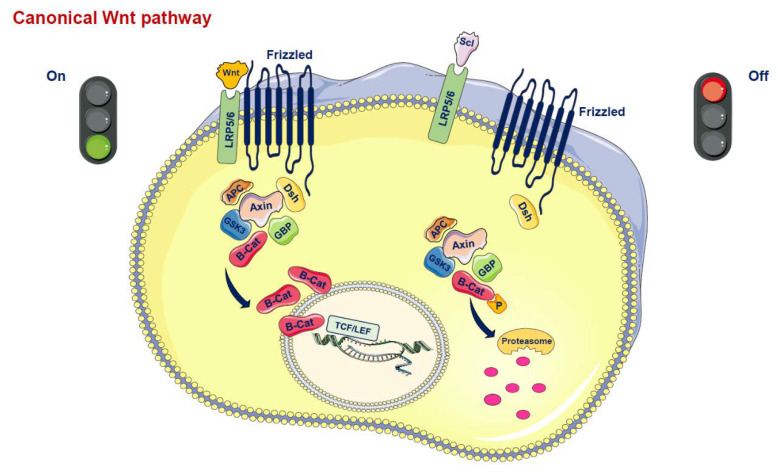
Canonical Wnt pathway. In the active state, Wnt ligands (Wnt) form a complex with the receptors low-density lipoprotein receptor-related protein 5 or 6 (LRP5/6) and Frizzled (Fz). Disheveled (Dsh) is then able to bind to Fz. Dsh forms a complex with glycogen synthase kinase 3ß (GSK3ß), GSK3 binding protein (GBP), axin and adenomatous polyposis coli (APC). This protects ß-catenin from proteasomal degradation. ß-catenin translocates into the nucleus and interacts with the T-cell factor/lymphoid enhancer factor (TCF/LEF) family of transcription factors to promote gene transcription. In the inactive state, inhibitors of this system, such as Sclerostin (SCL), prevent the formation of the Wnt-Fz-LRP5/6 complex. ß-catenin is degraded by proteasomes after GSK3ß-mediated phosphorylation, so the signal is stopped.

Wnt proteins are secreted glycoproteins that bind to the N-terminal extracellular cysteine-rich domain of the Frizzled (Fz) receptor family, which is characterized by a seven-transmembrane-span (Figure 1) [5,6,7,8]. In addition to the interaction between Wnt and the transmembrane Fz receptor, coreceptors are also necessary to promote Wnt signaling. Thus, the low-density lipoprotein-related protein 5/6 (LRP5/6) is required to mediate the canonical Wnt signal, forming a receptor complex after interaction with the Wnt protein [5]. The signal is transduced to the cytoplasmic phosphoprotein Dishevelled (Dsh/Dvl), which is able to directly interact with Fz. At this level of Dsh, the Wnt signal branches into at least three major cascades, i.e., the canonical pathway, the planar cell polarity (PCP) pathway and the Wnt/Ca^2+^ route. The hallmark of the canonical Wnt pathway is represented by the translocation of the cytoplasmic protein β-catenin into the nucleus. In the canonical pathway, the activated receptor signals through Dsh and acts on a protein complex containing axin, glycogen synthase kinase 3β (GSK3β), casein kinase 1α (CK1α) and adenomatosis polyposis coli (APC) molecules. This complex, in the absence of Wnt ligands, promotes the ubiquitination and degradation of β-catenin [5,7]. Conversely, the binding of Wnt ligands to the transmembrane Fz receptor and LRP5/6 coreceptor triggers the inactivation of the degradation complex leading to a translocation of β-catenin to the nucleus where it interacts with the T cell factor (TCF)/lymphoid-enhancer binding factor (LEF) and activates the transcription of Wnt target genes such as c-Myc, cyclin D1 and PPARδ, which govern cell growth as well as cell proliferation and survival [5].

Beyond this canonical pathway involving β-catenin, two other noncanonical pathways have been described: the Wnt/Ca^2+^ pathway, which affects cell adhesion and movement, and the noncanonical PCP, which mediates asymmetric cytoskeletal organization and the polarization of cells, all of which have been related to several human diseases [4,5,6].

As expected, the composite Wnt pathway is tightly regulated. A key level of its modulation occurs in the extracellular milieu due to a number of secreted Wnt antagonists including secreted frizzled related proteins (sFRPs) and the Wnt inhibitory factor-1 (WIF1), which bond directly to Wnt molecules and alter their ability to bind to the Wnt receptor complex. Another signalling modulation occurs via the Dickkopf (DKK) family members and sclerostin, which inhibit the pathway by binding to LRP5/6 [5,6,7,8,9]. Furthermore, the canonical and noncanonical Wnt signalling pathways are closely connected and cross-regulate each other in a Wnt signaling network [10,11].

## 3. Atherosclerosis and Wnt Signaling

Atherosclerosis is a multifactorial process during which circulating oxidized lipids accumulate in the subendothelial space and are subsequently internalized by resident macrophages, leading to the establishment of foam cells and finally of the atheromatic plaque core. As it is known, the development of atherosclerosis is associated with the migration and proliferation of vascular smooth muscle cells (VSMCs) and to endothelial activation, which are produced by multiple inflammatory pathways [12,13].

**Figure 2:**
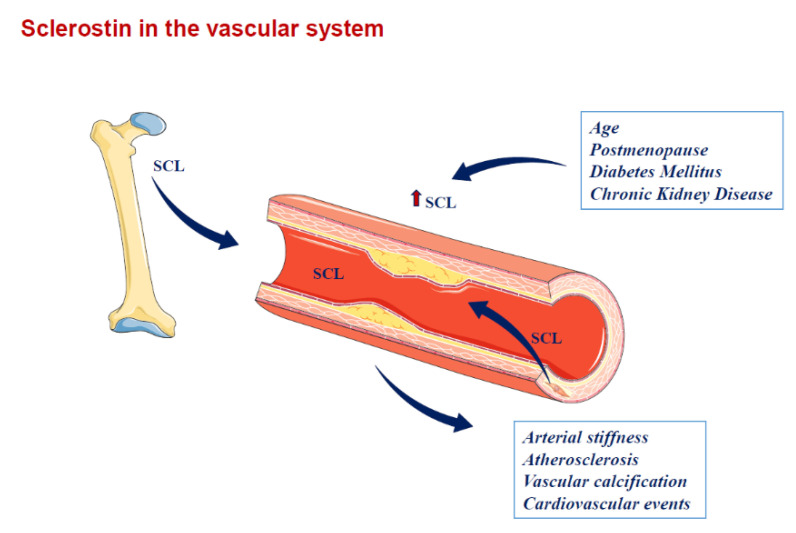
Sclerostin in the vascular system. SCL is produced by osteocytes at bone level and may drive the paracrine effect. SCL also spills over the circulation and may contribute to vascular pathophysiology (e.g., atherosclerosis, arterial stiffness and vascular calcification). SCL is also up-regulated in calcifying vascular smooth muscle cells at the vascular level. SCL = sclerostin. Arrows indicate the spill over of sclerostin from the sites where it is produced into circulation and the related factors or diseases associated with higher sclerostin circulating levels.

A consideration of Wnt signalling involvement in atherosclerosis was raised from certain clinical observations. In an Iranian family with early coronary artery disease, hypertension, hyperlipidemia and osteoporosis, a homozygous loss-of-function mutation R611C in the LRP6 gene was described in the affected subjects, along with an increased risk of carotid artery atherosclerosis in hypertensive subjects without hyperlipemia [14,15]. However, numerous study findings both from in vitro and in vivo investigations, as shown below, suggested contribution of Wnt signalling pathways to the vascular pathophysiology (Figure 2).

### Experimental Evidence of Wnt Pathway Involvement in the Atherosclerotic Process

Several researches exploring the pathophysiology of atherosclerosis focused on Wnt proteins as activators of the Fz receptor family (Table 1). High levels of Wnt5a have been detected in macrophage-rich areas of atherosclerotic plaques. Bacterial structures such as lipopolysaccharide (LPS) bind the Toll-like receptor 4 (TLR4), whose downstream signalling induces the expression of Wnt5a [16,17,18,19]. Wnt5a can promote the release of proinflammatory cytokines driving an inflammatory response. Contrariwise, Wnt3a, via the suppression of GSK3β, shows an anti-inflammatory effect which in turn modulates NFκB-dependent gene transcription [20].

Kin et al. observed that Wnt5a induces cyclooxygenase-2 expression and boosts the release of inflammatory cytokines. Particularly, they observed that a calcium ionophore enhances endothelial inflammation similarly, whereas calcium chelators and protein kinase C inhibitors block the Wnt5a-induced activation, suggesting a role of the Wnt/Ca^2+^/protein kinase C pathway in endothelial inflammatory regulation [21]. Furthermore, it has been observed that the Wnt antagonist DKK-1 increased in atherosclerotic plaques and was involved in the atherosclerotic plaque inflammatory response. Augmented levels of DKK-1 have been detected in experimental (ApoE(-/-) mouse) atherosclerosis and, as with the human clinical scenario, in patients suffering from coronary artery disease or from carotid plaques, both systemically and locally, with high levels noticed, above all, in advanced and unstable disease states. Moreover, a role for platelet- and endothelial-derived DKK-1 in platelet-dependent endothelial activation has been proposed due to its promotion of an inflammatory cytokine enhanced release [22].

Borrell-Pages et al. investigated the role of LRP5 in macrophage differentiation and migration upon lipid loading. LRP5 is transcriptionally regulated by aggregated low density lipoprotein (agLDL), participating in lipid uptake and transformation of macrophages into foam cells, a critical step in atherosclerosis progression. AgLDL-treated macrophages showed an upregulation of β-catenin, LEF1, c-jun, cyclin D1, bone morphogenetic protein 2 (BMP2) and osteopontin (OPN), proteins and targets of the Wnt signalling pathway, whereas LRP5-silenced macrophages showed a significant down-regulation of OPN and BMP2 expression. In addition, it was observed that LRP5-deficient macrophages exhibited impaired migration [23]. These findings suggest that LRP5 plays a role in the innate inflammatory response to lipid infiltration and thus takes part in the atherosclerotic process. 

VSMCs are capable of major phenotypic changes triggered by modifications of local clues including growth factors, mechanical stimuli, cell-cell and cell-matrix interactions and various inflammatory mediators. Migration of VSMCs from the media towards the intima is one of the hallmarks in the development of an atherosclerotic plaque. The activation of VSMCs leads to their proliferation, and migration is accompanied by a switch from a contractile towards a synthetic phenotype, which is characterized by an increased production of cytokines and extracellular matrix [24].

Wnt signaling via β-catenin is involved in the upregulation of proproliferative genes such as the cyclin D1 gene, which promotes VSMC proliferation [25].

Wnt1, Wnt3a and Wnt4 have been identified to induce activation of canonical Wnt signalling and cyclin D1 expression in VSMCs. On the other hand, the inhibition of Wnt signalling, via the overexpression of sFPR1, a member of the group of secreted frizzled related proteins (sFRP) expressed in the cardiovascular system, resulted in the inhibition of VSMC proliferation both in vitro and in vivo (Table 1) [26,27,28].

**Table 1:** Implication of Wnt related gene in atherosclerotic process.

Wnt Related Genes	Source	Effect	Mechanism	References
*Wnt1*	ND	VSMCs proliferation	cyclin D expression	[27,28]
*Wnt3a*	VSMCs, Macrophages	Anti-inflammatory	suppression of GSK3β	[20]
*Wnt4*	ND	VSMCs proliferation	cyclin D1 expression	[25,26]
*Wnt5a*	Macrophages	Release of pro-inflammatory cytokines	Activation of TLR4 Cyclooxygenase-2 expression	[16,17,18,19,21]
*DKK-1*	Platelets, endothelial cells	Release of pro-inflammatory cytokines	platelet-dependent endothelial activation	[22]
*sFRP1*	Cardiovascular system	Inhibition of VSMCs proliferation	Inhibition of Wnt signaling	[27]
*LRP5*	Macrophages	OPN and BMP2 expression; macrophages migration	Upregulation of c-jun and cyclin D1	[23]

## 4. Sclerostin in Atherosclerosis and Vascular Diseases

Sclerostin is encoded by the SOST gene, and SOST mRNA expression is detected in a wide number of organs including bone, cartilage, kidney, liver, pancreas, heart and also placenta and fetal skin. However, it has been shown that sclerostin in human subjects is mainly produced by osteocytes and cementocytes, and is identified in serum or plasma, even if, because of technical uncertainties regarding currently available sclerostin assays, circulating sclerostin levels should be interpreted cautiously [29,30].

Although the biological relevance of circulating protein measurements has not been fully elucidated, several clinical studies showed altered sclerostin and DKK-1 concentrations in metabolic bone diseases, in type 1 and 2 diabetes mellitus and in patients with chronic kidney disease (CKD) [31,32,33,34,35]. 

Interestingly, Pelletier et al. observed that serum sclerostin levels are higher in CKD patients compared with the general population and that they increase with the progression of CKD particularly during, or after, CKD stage III [36]. Thus, sclerostin is inversely correlated with glomerular filtration rate (GFR) [37,38,39]. The increased sclerostin serum levels observed in CKD could be caused by several factors including sclerostin renal retention, even if it has been reported that urinary sclerostin excretion rises with declining eGFR, but also with an enhanced production by bone cells [40,41].

Recently, SOST expression has been investigated at the vascular level. Particularly, a cross-sectional study involving 46 patients aged 55 to 80 years (mean 71.1 ± 6.7 years, 36 men, 15 patients with type 2 diabetes mellitus) explored sclerostin production in atherosclerotic plaques of patients who underwent carotid endarterectomy. Sclerostin was identified in the plaques of all the patients, and its levels were significantly higher in the media compared with the intima, as well as higher in VSMCs compared with infiltrating macrophages, irrespective of history of type 2 diabetes mellitus [42].

Sclerostin has been detected in the aorta of patients undergoing aortic valve replacement and is up-regulated in calcifying VSMCs and calcified valvular plaques [43,44,45,46]. It colocalized with vascular calcifications (VCs) of the media and its serum levels were significantly associated with the presence of thoracic aorta calcification (TAC), the severity of TAC and the positive expression of the SOST gene in the vascular system [47].

Furthermore, it should be mentioned that earlier analyses described associations between circulating sclerostin levels with aortic or carotid plaques and VCs, although the direction of association was inconsistent due to methodological issue [35,48,49,50]. These results were at least partially related to the instrumental detection of calcification, as some studies used vertebral x-ray scans to measure aortic artery calcification [48,49]. While this validated method is widely used by studies initially intended for osteoporosis research, it is a semi-quantitative scale with a lower reproducibility than CT-derived calcification measurements as detected through the Agatston method [50,51]. Additionally, previous studies also included different population samples such as diabetic patients, CKD patients or postmenopausal women [35,49,51] (Table 2).

As for the association with VCs, after adjusting for risk factors including age, physical and lifestyle characteristics, comorbidities, lipoproteins and kidney function, Kuipers et al. found that a sclerostin level greater than 1 SD was associated with a 1.61-times (95%CI 1.02–2.53) greater odds of having aortic artery calcification (CAC). However, sclerostin was not associated with aortic artery calcification (AAC) in any model, suggesting that sclerostin may differentially influence VCs in different vascular beds [50].

How can the correlation between sclerostin and VCs be interpreted? The positive association between serum sclerostin, a Wnt pathway inhibitor, with VCs may seem paradoxical at first sight. Since Wnt signalling is a driver of bone formation and enhances ectopic mineralization, a Wnt inhibitor would be associated with decreased mineralization. Indeed, an antisclerostin antibody treatment increased bone mineralization in humans, and subjects with mutations in the SOST gene showed bone overgrowth and increased mineralization [52,53,54]. It is noteworthy that sclerostin concentrations have been positively associated both with greater arterial calcification and consistently with greater bone mineral density [50,51].

It could be speculated that augmented serum sclerostin levels may be a physiological adaptation to increased calcification, and could even be a marker of some other mineralization pathway with which the Wnt pathway interacts, such as the nuclear factor kB (RANK)/RANK ligand/osteoprotegerin (OPG) pathway which is also associated with vascular disease in humans [55,56].

Krishna et al. studied the role of SOST in aortic aneurysm (AA) and atherosclerosis using human samples, a mouse model and in vitro experiments. Their research identified that SOST is expressed in the aorta and downregulated in human AA, possibly because of epigenetic silencing. They reported that SOST inhibits angiotensin II (AngII)-induced AA in both the thoracic and abdominal aorta of the mouse model, and also inhibits AngII-induced atherosclerosis. Therefore, upregulation of SOST inhibits AA and atherosclerosis development. These findings suggested a potential implication in the treatment of some vascular diseases [57].

Sclerostin has been proved to be linked with subclinical atherosclerosis. In fact, it was positively or inversely associated with carotid intima-media thickness (CIMT) in postmenopausal women suffering from type 2 diabetes mellitus [49,58].

Looking at patients with CKD, in nondialysis subjects DKK-1, but not sclerostin, was inversely associated with arterial stiffness [58].

Sclerostin serum levels have also been explored in hemodialysis (HD) patients. In a retrospective study, subjects with sclerostin levels above the median value showed a higher prevalence of atherosclerotic plaques (*p* = 0.025) and a higher CIMT (*p* = 0.038). Moreover, at the median follow-up of 61.2 months, high sclerostin baseline levels were evocative of shorter survival predictors of all-cause mortality [59].

Noteworthy, a significant negative association between higher circulating sclerostin levels with mortality was observed in a prospective cohort study of incident dialysis patients (*n* = 673, age 63 ± 14 yrs.), in the Netherlands. Particularly, after adjustment for various clinical and biochemical parameters, patients in the highest sclerostin tertile had a significantly lower risk of cardiovascular death [hazard ratio 0.29, 95% confidence interval (CI) 0.13–0.62] and for all-cause mortality (0.39, 95% CI 0.22–0.68) within 18 months compared with patients of the lowest tertile. The association of sclerostin levels with outcome was less pronounced for long-term cardiovascular mortality and absent for noncardiovascular mortality [60].

In addition, in a study based on 207 HD patients, subjects in the tertile of higher sclerostin levels, when compared with the tertile of lower sclerostin levels, were significantly older (73.7 ± 12 vs. 64.7 ± 18 yrs.), more frequently of the male gender (74 vs. 48%), had lower serum bone-specific alkaline phosphatase values (14 ± 9 vs. 20.4 ± 13 µg/L), were less frequently treated with alfacalcidol, displayed lower Kauppila aortic calcification scores (9.5 ± 5 vs. 12.5 ± 7/24), had higher bone mineral density (BMD) and showed a lower all-cause mortality rate during a 30-month follow-up period at a multivariable adjusted Cox model (hazard ratio 0.5, 95% CI 0.25–0.93, *p* = 0.03) [61].

However, in a cohort of 165 dialysis patients (mean age 56.5 ± 15.6; 84 hemodialysis (HD) and 81 peritoneal dialysis (PD)), during the median follow-up period of 24.9 months, sclerostin level was only an independent predictor of all-cause mortality and cardiovascular events (CVEs) in patients with PD after adjusting for confounding factors (*p* < 0.05). Low serum sclerostin was associated with a better overall survival and a lower prevalence of CVEs in patients with PD, but had no relationships in patients with HD [62]. Furthermore, in a predialysis CKD cohort, higher serum sclerostin values were associated, even after multiple adjustments, with fatal and nonfatal CVEs and mortality [63].

Nevertheless, when exploring the effect of sclerostin on CVEs, all-cause/cardiovascular mortality and VCs in patients with CKD, contradictory data exist mainly due to heterogeneity of participants and observation periods. Consequently, in a systematic review and meta-analysis, including nine observational prospective studies involving 1788 patients, an association between sclerostin levels and development of fatal and nonfatal CVEs and all-cause mortality in CKD was not observed [64].

As it is generally known, arterial stiffness describes the rigidity of the arterial wall and is a hallmark of arterial aging [13,48]. In a previous study designed to investigate the relationship between serum levels of sclerostin and DKK-1 with CIMT and arterial stiffness measured by pulse wave velocity (PWV), a predictor of cardiovascular and all-cause mortality, sclerostin, but not DKK-1, emerged as an independent predictor of arterial stiffness in healthy adult outpatient subjects after correcting for confounders [65]. The discrepancy in the behaviour of these two inhibitors of Wnt signalling, which play almost the same role in the vascular cells, has been reported in other studies and may be accounted for by the widespread expression of DKK-1 compared with that of sclerostin [58,66,67].

Sclerostin serum levels were reported to be positively associated with carotid-femoral PWV in several cohorts including women with postmenopausal osteoporosis and patients with CKD [48,66,67].

In a group of 154 HD patients, Jin S. et al. examined the relationship between circulating sclerostin levels and carotid-femoral PWV as a marker of arterial stiffness. They found that serum sclerostin levels were higher in patients with arterial stiffness, but such correlation was lost after adjustments for age, blood pressure, mineral and lipid parameters in a multivariate analysis. However, in a subgroup analysis, according to categories of parathyroid hormone (PTH) serum levels, serum sclerostin level appeared as a significant independent predictor for carotid-femoral PWV in patients with a PTH level under 300 pg/mL, with sclerostin possibly being a suitable biomarker for arterial stiffness [68]. PTH has been observed to inhibit the expression of sclerostin [69,70]. Thus, in HD patients with high PTH levels, continuous elevated PTH may suppress the expression of the SOST gene, reducing the secretion of sclerostin and consequently leading to the loss of its hypothetical defensive cardiovascular effect (Figure 2).

Similar to arterial stiffness, VCs are recognized as strong predictors of all-cause and cardiovascular mortality in CKD patients. VCs have been consistently associated with a number of traditional risk factors including age, hypertension and diabetes, as well as with nontraditional risk factors including mineral metabolism disorders [35].

Hampson et al. investigated the association between circulating concentrations of Wnt inhibitors, DKK-1 and sclerostin with bone mineral density (BMD), AAC and arterial stiffness in a cohort of 146 post-menopausal women [48]. AAC, detected by vertebral fracture assessment (VFA) imaging, was quantified using a previously validated 24-point scoring system demonstrating the extension of AAC at the site adjacent to the lumbar vertebrae L1-L4. By dividing the participants into 2 groups based on a PWV cut-off value of 9 m/s, which was representative of the mean value observed in post-menopausal women, they found that serum sclerostin was significantly higher in women with a PWV > 9 m/s compared to women with a PWV < 9 m/s. After adjustment for age, BMI, smoking habits, blood pressure and lipid levels, a positive association was observed between sclerostin and the AAC score. Differently, DKK-1 showed a negative association with AAC, probably due to a different regulation of its production, not only by osteogenic VSMCs, but also by endothelial cells, platelets or inflammatory cells within the atherosclerotic plaque, which may contribute to circulating DKK-1 concentrations [48]. Therefore, low concentration of DKK-1 could promote mineralization and VCs, whereas high sclerostin levels could attenuate advancing VCs. An equilibrium between these two Wnt inhibitors controls the balance of vascular biology and calcification process.

Focal intimal calcification of atherosclerotic plaques is a common finding that can affect plaque stability and increase the risk of plaque rupture. Conversely, medial calcification is often widespread and leads to increased arterial stiffness, left ventricular hypertrophy and cardiovascular events. Both forms of arterial intimal and medial calcification of arterial wall enhance mortality risk [35,48,65].

In a cohort of CKD patients undergoing HD, with a median age of 53 years, Pelletier et al. investigated the association between sclerostin and bone status evaluated by high-resolution peripheral quantitative computed tomography (HR-pQCT) at the tibial site, and AAC assessed according to the Kauppila method on lateral spine imaging using dual-energy x-ray absorptiometry (DXA). They found that higher sclerostin serum levels and poorer tibia cortical thickness were positively and independently associated with higher odds of severe AAC (Kauppila score of 6 and above). Accordingly, they proposed sclerostin as one of the players involved in the association of mineral and bone disorder with VCs in HD patients [71]. Sclerostin is thus a potential missing link between bone and vascular systems, and participates in the vascular homeostasis. 

It has been reported that several drugs may interfere with sclerostin levels mainly related to the treatment of osteoporosis (e.g., antiresorptive and anabolic drugs), but few data are available as to the clinical vascular outcomes and much more focused studies are needed [34,69,70,72,73,74,75].

Recently, concerns about cardiovascular safety were raised following the development of a new antiosteoportic treatment based on the monoclonal antibody Romosozumab. It works by inhibiting the activity of sclerostin, leading simultaneously to increased bone formation and, to a lesser extent, decreased bone resorption [76,77]. A recent systematic review and meta-analysis proved that Romosozumab therapy does not increase the risk of composite cardiovascular outcomes (1.26 [95% CI, 0.95–1.68], *p* = 0.11), and three-point major adverse cardiovascular events (1.41 [95% CI, 0.99–2.02], *p* = 0.06), while it increases the risk of four-point major adverse cardiovascular events (1.39 [95% CI, 1.01–1.90], *p* = 0.04) among elderly men and postmenopausal women with osteoporosis over a period of 12–36 months. It has also been highlighted that Romosozumab does not increase or reduce specific cardiovascular outcomes including CV death, myocardial infarction, stroke, atrial fibrillation, heart failure, aortic and intracranial aneurysm, aortic dissection, aortic valve disease and hypertension [78].

In conclusion, several lines of evidence suggest the relevant contribution of sclerostin in the pathophysiology of vascular homeostasis. Sclerostin is a specific and regulated modulator of the Wnt pathway working in several tissues and particularly in bone and vessels. Aging is accompanied by physiological and morphological changes in the cardiovascular system, sometimes reflecting modification of mineral and bone metabolism. Regulators of Wnt signalling, and particularly sclerostin, could become future markers of bone-vessel cross-talk and, possibly, could be included in diagnostic cardiovascular work-up. Drug-induced modulation of sclerostin may even imply subclinical or clinical cardiovascular consequences.

**Table 2:** Association between sclerostin levels and cardiovascular events.

Sclerostin Levels	Cardiovascular Events	References
↑	↓ CIMT	[58]
↑	↑ prevalence of atherosclerotic plaques and ↑ CIMT	[49,59]
↑	↑ PWV	[48,65,66,67,68]
↑	↑ AAC	[48,71]
↑	↓ all-cause mortality and CVEs	[60]
↑	↑ AAC; ↑ all-cause mortality	[61]
↑	↑ all-cause mortality and CVEs	[62]
↑	↑ fatal and non fatal CVEs	[63]

↑ = higher sclerostin levels; ↓ = lower sclerostin levels; CIMT = carotid intima-media thickness; PWV = pulse wave velocity; CVEs = cardiovascular events; AAC = aortic artery calcification.

## Abbreviation List

AA	aortic aneurysm
AAC	aortic artery calcification
agLDL	aggregated LDL
AngII	angiotensin II
APC	adenomatosis polyposis coli
ApoE	apolipoprotein E
BMI	body mass index
BMD	bone mineral density
BMP2	bone morphogenetic protein 2
Ca^2+^	calcium
CAC	coronary artery calcification CAC = coronary aortic calcification
CK1α	casein kinase 1α
CIMT	carotid intima-media thickness
CKD	chronic kidney disease
CVEs	cardiovascular events
DXA	dual-energy x-ray absorptiometry
DKK-1	Dickkopf-related protein 1
Dsh	dishevelled
Fz	frizzled
GFR	glomerular filtration rate
GSK3β	glycogen synthase kinase 3β
HD	hemodialysis
HR-pQCT	high-resolution peripheral quantitative computed tomography
LEF	lymphoid-enhancer binding factor
LDL	low-density-lipoprotein
LRP5/6	low-density-lipoprotein-related protein5/6
LPS	lipopolysaccharide
NFκB	nuclear factor κB
OPG	osteoprotegerin
OPN	osteopontin
PCP	planar cell polarity
PD	peritoneal dialysis
PPARδ	peroxisome proliferator-activated receptor δ
PTH	parathyroid hormone
PWV	pulse wave velocity
RANK	receptor activator of nuclear factor kappa-Β
RANKL	receptor activator of nuclear factor kappa-Β ligand
sFRP	secreted frizzled related proteins
TAC	thoracic aorta calcification
TCF	T cell factor
TLR4	toll-like receptor 4 agonist
VCs	vascular calcifications
VFA	vertebral fracture assessment
VSMCs	vascular smooth muscle cells
WIF	Wnt inhibitory factor-1
Wnt	wingless-type MMTV integration site family
